# Homozygous sequence variants in the *WNT10B* gene underlie split hand/foot malformation

**DOI:** 10.1590/1678-4685-GMB-2016-0162

**Published:** 2018-01-22

**Authors:** Asmat Ullah, Ajab Gul, Muhammad Umair, Irfanullah, Farooq Ahmad, Abdul Aziz, Abdul Wali, Wasim Ahmad

**Affiliations:** 1 Department of Biochemistry, Faculty of Biological Sciences, Quaid-i-Azam University, Islamabad, Pakistan.; 2 Department of Biotechnology and Informatics, BUITEMS, Quetta, Pakistan.; 3 Department of Computer Sciences and Bioinformatics, Khushal Khan Khattak University, Karak, Pakistan.

**Keywords:** Split-Hand-Foot Malformation 6, WNT10B gene, sequence variants

## Abstract

Split-hand/split-foot malformation (SHFM), also known as ectrodactyly is a rare genetic disorder. It is a clinically and genetically heterogeneous group of limb malformations characterized by absence/hypoplasia and/or median cleft of hands and/or feet. To date, seven genes underlying SHFM have been identified. This study described four consanguineous families (A-D) segregating SHFM in an autosomal recessive manner. Linkage in the families was established to chromosome 12p11.1–q13.13 harboring *WNT10B* gene. Sequence analysis identified a novel homozygous nonsense variant (p.Gln154*) in exon 4 of the *WNT10B* gene in two families (A and B). In the other two families (C and D), a previously reported variant (c.300_306dupAGGGCGG; p.Leu103Argfs*53) was detected. This study further expands the spectrum of the sequence variants reported in the *WNT10B* gene, which result in the split hand/foot malformation.

## Introduction

Split-hand/split-foot malformation (SHFM) is a congenital limb defect characterized by absence of medial digital rays, median clefts of the hands and feet, aplasia and/or hypoplasia of the phalanges, and syndactyly (McKusick, 1998; Duijf *et al.*, 2003). In severe cases, feet exhibit a lobster claw-like appearance with a deep median groove. Asymmetric manifestations, variable syndactyly and nonpenetrance of the disorder have also been described (Ozen *et al.*, 1999). Few SHFM patients have been found with signs of intellectual disability, ectodermal and craniofacial defects, and orofacial clefting (Elliott and Evans, 2006). Clinical features of SHFM vary from patient to patient and within the same individual (Duijf *et al.*, 2003; Elliott and Evans 2008; Sowinska-Seidler *et al.*, 2014). Both isolated (non-syndromic) and syndromic forms of SHFM have been reported in the literature.

Seven types of non-syndromic SHFM have been mapped on different human chromosomes. Four of these forms (SHFM-1, SHFM-3, SHFM-4, SHFM-5) inherit in autosomal dominant pattern, SHFM-2 in X-linked inherited, and SHFM-6 in autosomal recessive pattern. The four autosomal dominant forms were mapped on chromosome 7q21 (SHFM-1; MIM183600) (Scherer *et al.*, 1994), 10q24 (SHFM-3; MIM246560) (Nunes *et al.*, 1995; Gurrieri *et al.*, 1996; Raas-Rothschild *et al.*, 1996), 3q27 (SHFM-4; MIM605289) (Ianakiev *et al.*, 2000) and 2q31 (SHFM-5; MIM606708) (Boles *et al.*, 1995). The autosomal recessive form (SHFM-6; MIM 225300) has been mapped on chromosome 12q13.11-q13 (Ugur and Tolun, 2008). The X-linked form was mapped on Xq26, which was later refined to a small region of 5.0 cM (Faiyaz-Ul-Haque *et al.*, 2005). Based on genome-wide linkage analysis in a single family with autosomal recessive inheritance pattern, Gurnett *et al.* (2006) mapped a novel locus for SHFM on chromosome 8q21.11–q22.3. For these seven loci, only three (SHFM1, SHFM4, SHFM6) have been solved at the gene level. A causative gene of the wingless-type MMTV integration site family, member 10 (*WNT10B*, MIM 601906) has been reported of causing SHFM-6 (Ugur and Tolun, 2008). Ianakiev *et al.* (2000) reported the gene *TP63* (tumor protein p63, MIM 603273), which encodes a homolog of the tumor suppressor responsible for the SHFM-4 phenotype. Shamseldin *et al.* (2012) reported a mutation in the gene *DLX5* responsible for the SHFM-1 phenotype in a consanguineous family. We reported intragenic variants in the *DLX5* and *DLX6* genes that caused the SHFM-1 phenotype in two different families (Ullah *et al.*, 2016a,b). Goodman *et al.* (2002) proposed that SHFM-5 and other digit defects may be caused by haploinsufficiency of genes (*EVX2*, *DLX1*, *DLX2*) located at the 5′ end of the *HOXD* cluster. Recently, Umair *et al.* (2017) reported a homozygous variant in the *EPS15L1* gene located on chromosome 19p13.11 causing isolated SHFM in a Pakistani family.

In this study, we investigated four consanguineous families of Pakistani origin showing split hand and foot malformation phenotypes. Linkage in the families was established to SHFM6 on chromosome 12p11.1-q13.13. Sequence analysis of the *WNT10B* gene detected a novel nonsense and a previously reported variant in affected individuals.

## Materials and Methods

### Family history

**Figure 1:**
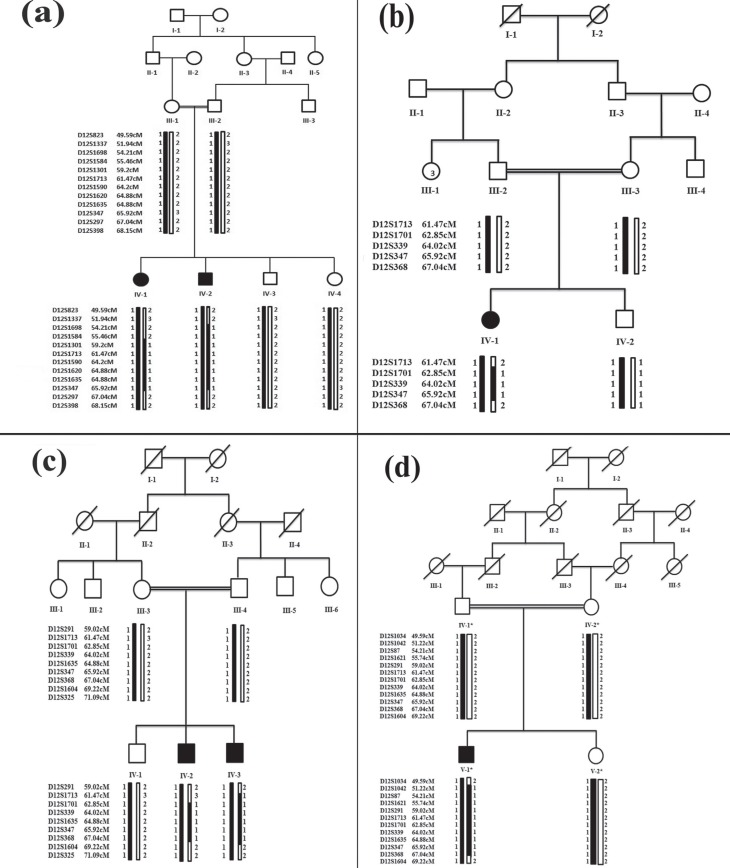
Haplotypes constructed in families (A to D) segregating SHFM6. Black symbols represent affected individuals while clear symbols represent unaffected individuals. The shaded black alleles are forming risk haplotype, while the alleles shown in white are not co-segregating with the disease. Genetic distances in centi-Morgans (cM) are according to the Rutgers combined linkage-physical map (Build 36.2).

Approval to conduct the study was obtained from the Institutional Review Board (IRB) of Quaid-i-Azam University, Islamabad Pakistan. Four consanguineous families with clinical manifestations of split hand and foot malformations (SHFM were recruited from remote areas of Pakistan. Pedigree reconstruction (Figure 1) provided convincing evidence of an autosomal recessive mode of inheritance of the disorder. Six individuals with SHFM were identified in four families. This included a male (IV-1) and a female (IV-2) in family A, a female (IV-1) in the fourth generation in family B, two males (IV-2, IV-3) in the fourth generation in family C, and one male (V-1) in the fifth generation in family D. All affected and unaffected individuals in the four families were examined by medical specialists at local government hospitals. Blood samples were collected from both affected and unaffected individuals of the families after obtaining written consent.

### Isolation of Genomic DNA

Peripheral blood samples were obtained from 19 individuals in EDTA containing vacutainer sets (BD, Franklin Lakes, NJ, USA). Genomic DNA extraction was performed using a standard phenol-chloroform procedure. DNA was quantified using a Nanodrop-1000 spectrophotometer (Thermal Scientific, Wilmington, MA).

### Genotyping

Linkage in the families was searched by genotyping microsatellite markers mapped in the flanking regions of autosomal dominant and autosomal recessive forms of SHFM. This included SHFM1 (D7S2537, D7S2481, D7S630, D7S492, D7S627, D7S1813, D7S657, D7S527, D7S479) at chromosome 7q21, SHFM3 (D10S520, D10S91, D10S1736, D10S1726, D10S603, D10S1710, D10S383, D10S1264) at chromosome 10q24, SHFM4 (D3S3570, D3S3600, D3S3596, D3S1661, D3S2747, D3S1662, D3S2311, D3S1305) at chromosome 3q27, SHFM5 (D2S124, D2S2345, D2S294, D2S2302, D2S1274, D2S2257, D2S2173, D2S2978) at chromosome 2q31, SHFM6 (D12S1034, D12S823, D12S1042, D12S1337, D12S1698, D12S87, D12S1584, D12S1621, D12S291, D12S1301, D12S1713, D12S1701, D12S339, D12S1590, D12S1620, D12S1635, D12S347, D12S297, D12S368, D12S398, D12S1604, D12S325) at chromosome 12q11-q13, and another SHFM locus mapped on chromosome 8q21.11–q22.3 (D8S526, D8S2321, D8S1119, D8S1818, D8S1129, D8S1714, D8S556) (Gurnett *et al.*, 2006). PCR amplification of the microsatellite markers was performed as previously described (Ullah *et al.*, 2015). The amplified PCR products were resolved on 8% non-denaturing polyacrylamide gels, stained with ethidium bromide, and genotypes were assigned by visual inspection. DNA ladders of 5, 10 and 20 bp (MBI Fermentas®, Life Sciences, York, UK) were used to determine allele size for respective microsatellite markers. Markers used in the genotyping were arranged according to Rutgers combined linkage-physical map (Build 36.2) of the human genome (Matise *et al.*, 2007). Haplotypes were analyzed by SIMWALK2 (Sobel and Lange, 1996).

### Sequencing of the WNT10B gene

Primers used for PCR amplification, sequencing and coding of intron-exon junctions of the *WNT10B* gene were the same as described earlier (Khan *et al.*, 2012). The PCR-amplified products were purified with a commercially available kit (Axygen MD, USA) and sequenced using ABI BigDye Terminator Sequencing Kit v.3.1 (Applied Biosystems, Foster City, CA, USA). Sequence variants were identified via the BIOEDIT sequence alignment editor, version 6.0.7 (Ibis Biosciences, CA, USA).

### *In silico* analysis

The pathogenicity index of the sequence variants identified here was calculated using the following softwares: Mutation Taster (http://www.mutationtaster.org/), Polymorphism Phenotyping V2 (PolyPhen-2) (http://genetics.bwh.harvard.edu/pph2/) and Sorting Intolerant From Tolerant (SIFT) (http://sift.bii.a-star.edu.sg/). The frequency of the variants in the general population was determined using the Exome Variant Server (EVS) (http://evs.gs.washington.edu/EVS/), and 1000 genomes.

## Results

### Clinical features

**Figure 2:**
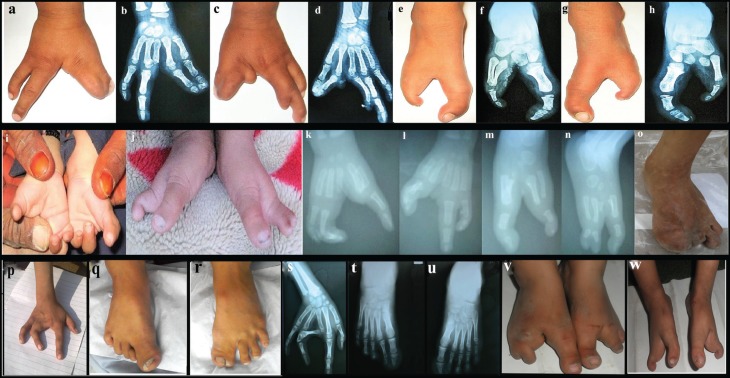
Clinical features of split hand/foot malformation (SHFM) observed in family A. (a) Female patient (IV-1) showing cleft hand deformity with absence of middle finger in right hand, and syndactyly of 1^st^ and 2^nd^ finger. (b) Radiograph showing pre-axial syndactyly of index finger and thumb at distal phalanx as well as in 3^rd^ and 4^th^ metacarpals. (c, d) The same patient (IV-1) showing cleft hand deformity with central type syndactyly in her left hand, and with an additional bud on the 2^nd^ metacarpal; distal phalanx in the middle finger is missing. (e, h) Classical cleft feet deformities characterized by central deficiency in the same affected individual, radiographs showing hallux valgus deformities in the big toe, missing central toes and presence of postaxial syndactyly in the metatarsal bones; some of the tarsal bones are missing. (i, j) Male affected individual (IV-2) showing cleft hand deformity associated with missing of 2^nd^ and 3^rd^ finger. (k, n) The same affected member showing cleft feet deformity; radiological study showing bilateral cleft hand/feet deformities associated with preaxial and postaxial syndactyly in the hands and feet, respectively. (o) Clinical features of an affected girl (IV-1) in family B showing missing big toe along with 2^nd^ and 3^rd^ toes. (p) An affected individual (IV-2) in family C showing absence of middle phalanx of 3^rd^ finger in right hand, severe bilateral hypoplasia and fusion of the 3^rd^ and 4^th^ finger. (q, r) The same patient, showing pre-axial syndactyly in toes 1 and 2 in both feet, and an additional bud on the 3^rd^ toe in right foot. (s) Radiographic features of the right hand of the patient (IV-2) showing radial ray malformation including hypoplasia of 1^st^ metacarpal, complex fusion of middle finger to ring finger, relatively large proximal phalanx of 5^th^ finger, fingers showing contractures and deviations. (t-u) Radiographs of the feet showing fusion of the 1^st^ and 2^nd^ toes. (v) Affected individual (IV-3) in family C showing bilateral cleft foot with missing 2^nd^ and 3^rd^ digits, central deficiency with rudimentary bud of lesser toe, classical cleft foot, and basal syndactyly formation. (w) Affected individual (V-1) in family D showing cleft foot deformity, i.e. longitudinal deficiency of digital ray of the foot except rays 1 or 5, central deficiency and varus deformity of lesser toe, claw toe deformity, classical cleft foot and fusion of the middle and distal phalanx of 5^th^ toe.

Affected individuals in the four families showed classical phenotypes of SHFM, which segregated in an autosomal recessive manner. At the time of the study, ages of the patients varied between 2 to 10 years. Patients were born after normal pregnancies. All the affected individuals showed cleft hand and cleft foot deformities associated with mesoaxial syndactyly (Figure 2).

In family A, the affected female (IV-1) showed cleft hand deformity with absence of the middle finger in the right hand. Radiographs showed pre-axial syndactyly of the index finger and thumb at the distal phalanx as well as in the 3rd and 4th metacarpals (Figure 2a,b). The same patient showed cleft hand deformity with central syndactyly in her left hand with an additional bud on the 2nd metacarpal. The distal phalanx of the middle finger was also missing (Figure 2c,d). Classical cleft feet deformities, characterized by central deficiency, were present. Radiographs showed hallux valgus deformities of the big toe and missing central toes associated with postaxial syndactyly in the metatarsal bones. Some of the tarsal bones were absent (Figure 2e-h). The male individual of the same family (IV-2) showed the same SHFM phenotypes as the female (IV-1). Abnormalities such as syndactyly, radial ray malformation, dysplastic hands, and cleft feet were noted (Figure 2i-j). The radiological study revealed bilateral cleft hand/feet deformities associated with pre-axial and postaxial syndactyly in hands and feet, respectively (Figure 2k-n).

In family B, an affected girl (IV-1) was missing the big toe and toes 2 and 3 in the right foot. The left foot and the hands were apparently normal (Figure 2o).

One of the affected members (IV-2) of family C showed aplasia of the middle phalanx of the 3rd finger in the right hand, severe bilateral hypoplasia and fusion of the 3rd and 4th finger (Figure 2p). Fusion of the big toe and 2nd toe in both feet, and an additional rudimentary bud were observed in the 3rd toe of the right foot. Radial ray malformation including hypoplasia of the 1st metacarpal, as well as complex fusion of the middle and ring fingers. Fingers with contractures and deviations were observed in radiographs of the right hand of patient IV-2 (Figure 2q-u). The other affected member (IV-3) of family C showed bilateral cleft foot with missing 2nd and 3rd toes, characterized by central deficiency with rudimentary bud of lesser toe, classical cleft foot, and basal syndactyly formation (Figure 2v).

In family D, the affected person (V-1) showed cleft foot deformity, i.e. longitudinal deficiency of the digital ray of the foot except rays 1 and 5, central deficiency and varus deformity of the lesser toe, claw toe deformity, classical cleft foot and fusion of middle and distal phalanx of the 5th toe (Figure 2w). Abnormalities in skin, teeth, face, nails, and eyes were not observed in affected members of the four families. Parents of the affected people were normal and healthy.

### Linkage and mutation analysis

**Figure 3:**
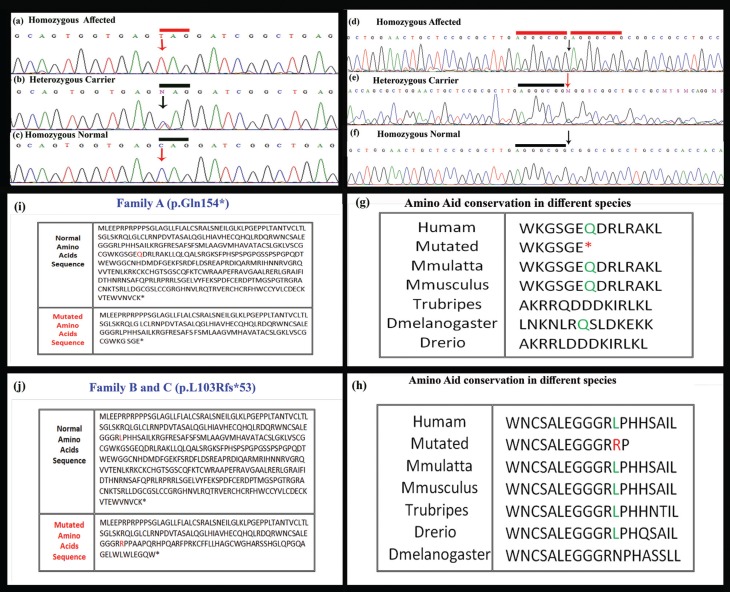
Sequence analysis of *WNT10B* gene showing a novel nonsense variant (c.460C > G, p.Gln154*) in affected individuals in family A and B. The upper panel (a) represents nucleotide sequences in an affected individual, the middle panel (b) in a heterozygous carrier and lower panel (c) in a normal individual; arrows indicate position of the sequence variant. (d) 7-bp duplication mutation (c.300_306dupAGGGCGG; L103Rfs*53) in the gene *WNT10B* found in affected individuals in family C and D, (e) in the heterozygous carrier, and (f) in an unaffected member of the family. Underline 7-bp sequence AGGGCGG is duplicated in affected members; arrows indicate position of duplication. (g, h) Leucine (L) amino acid represented in green is conserved across four different species. (i) Comparison of amino acid sequence of normal and mutated human WNT10B protein showing nonsense mutation (p.Gln154*) identified in family A and B. (j) Amino acid sequence comparison of normal and mutated human WNT10B protein showing substitution of leucine with arginine at amino acid position 103 and frame shift (Leu103Argfs*53). Comparison of amino acid sequence of human WNT10B protein with other orthologs.

Linkage in the families was tested by genotyping microsatellite markers. Haplotypes revealed that affected individuals were homozygous for markers linked to the *WNT10B* gene on chromosome 12q11-q13 (Figure 1). Subsequently, the *WNT10B* gene was sequenced in affected and unaffected members of the families. Sequence analysis detected a novel homozygous nonsense variant (c.460C > G, p.Gln154*) in exon 4 of the *WNT10B* gene in all the three affected members of the families A and B. In the two other families (C and D) a 7 bp duplication mutation (c.300_306dupAGGGCGG; p.Leu103Alafs*53) was detected in the affected members (Figure 3). The two sequence variants were present in heterozygous state in the obligate carriers in the families and were not identified in 150 ethnically matched control individuals. This absence and the available databases of genetic variations (ExAc, 1000 genome, EVS or dbSNP) confirm that these were not polymorphisms.

## Discussion

To date, five sequence variants in the *WNT10B* gene causing SHFM have been reported. Clinical findings resulting from these variants are summarized in Table 1. Ugur and Tolun (2008) reported a large consanguineous family of Turkish origin in which affected individuals were homozygous for p.Arg332Trp in the *WNT10B* gene. Blattner *et al.* (2010) found a 4 bp duplication (c.458_461dupAGCA) in a Swiss woman affected with sporadic SHFM6. Previously, four families of Pakistani origin carrying variants in the *WNT10B* gene have been reported. This included a missense variant (p.Thr329Arg) (Khan *et al.*, 2012), 4 bp deletion (c.1165_1168delAAGT), and 7 bp duplication (c.300_306dupAGGGCGG) (Aziz *et al.*, 2014). A family reported by Khan *et al.* (2012) showed absence of fingers and thumbs, which were not observed in the families studied by Ugur and Tolun (2008) and Blattner *et al.* (2010).

**Table 1:** Comparison of the clinical features, due to sequence variants in the *WNT10B* gene, observed in participants of the present study and those reported previously.

Mutation	Phenotypes	Reference
p.Arg332Trp	Syndactyly type I, postaxial partial syndactyly, flexion deformity of finger 2, Postaxial syndactyly (fingers 3–4) with almost fused nail beds, finger 5 clinodactyly, polydactyly type 1.	Ugur and Tolun (2008)
p.Thr329Arg	Syndactyly of radial ray and a rudimentary finger, pre-axial syndactyly of toe 1–2, post-axial syndactyly of toe 3–4 complex duplication of digit 3–4, Post-axial syndactyly of toe 3–4, absence of toe 2, digit 2 hypoplasia, agenesis of radial ray along with digit 1, pre-axial polydactyly type 1, Agenesis of distal ray at metacarpophalangeal joint level, fixed flexion contracture of both 4–5 digit at proximal interphalangeal joint level.	Khan *et al.* (2012)
p.Lys388Glufs*36, p.Leu103Argfs*53	Central and pre- axial syndactyly, campodactyly, polydactyly, dysplastic (abnormal development) hands and cleft feet, hallux valgus deformity of big toe and rudimentary bud of lesser toes.	Aziz *et al.* (2014)
p.Gln154*	Mesoaxial type of syndactyly, pre-axial syndactyly of index finger and thumb at distal phalanx, cleft hand deformity with absence of middle finger, Aplasia of distal phalanx of the middle finger, hallux valgus deformities of big toe, missing central toes, missing of great thumb	Present study
p.Leu103Argfs*53	Syndactyly of the great thumb and 2^nd^ toe in feet and an additional rudimentary bud on 3^rd^ toe, hypoplasia of 1^st^ metacarpal, complex fusion of middle to ring finger, aplasia of middle phalanx of 3^rd^ finger, bilateral hypoplasia and fusion of the 3^rd^ and 4^th^ finger, fingers with contractures and deviations, central deficiency and varus deformity of lesser toe, claw toe deformity.	Present study

In the present study, we have reported four additional Pakistani families with SHFM6 in autosomal recessive pattern. The SHFM features observed in affected members of the families included syndactyly, cleft hand/foot malformation, hallux valgus deformities, aplasia, hypoplasia, radial ray malformation, presence of extra rudimentary bone, hypoplastic finger and missing of phalanges. Features observed in affected members of the four families were similar to those reported previously (Ugur and Tolun, 2008; Khan *et al.*, 2012; Aziz *et al.*, 2014). However, polydactyly in few affected members of the families reported by Khan *et al.* (2012) and Aziz *et al.* (2014) were not observed in our study.

Microsatellite-based genotyping established linkage in the four families to SHFM6 on chromosome 12q11-q13, harboring the *WNT10B* gene. The gene *WNT10B* contains five exons spanning 6.5 kb of genomic DNA and gives rise to a 389 amino acids protein. WNT10B is a 45 kDa glycoprotein that plays a role in fetal limb bud development and adult hematopoiesis. Sequence analysis of the gene led to the identification of a novel nonsense variant (p.Gln154*) in two families (A and B) and a previously reported duplication (c.300_306dupAGGGCGG; p.Leu103Argfs*53) in the other families (C and D). The sequence variant (p.Gln154*) identified in family A and B is the first nonsense and sixth variant detected in the *WNT10B* gene. The premature stop codon at position 154 (p.Gln154*) results in a truncated transcript, which is probably degraded by nonsense mediated mRNA decay (Maquat, 1996, 2005). The 7 bp duplication (c.300_306dupAGGGCGG; Leu103Argfs*53) found in the other two families (C, D) changes the downstream nucleotide sequence, resulting in a premature stop codon (TGA). Thus, the mutant transcript produced is probably also degraded by nonsense-mediated mRNA decay.

The gene *WNT10B* belongs to the WNT gene family. Proteins encoded by the WNT gene family are involved in the activation of a canonical signaling pathway, acting as ligands for cell surface receptors complexes composed of frizzled (FZ) and low-density lipoprotein receptor-related protein 5/6 (LRP5/6) family members. Gordon and Nusse (2006) reported that binding of WNT to FZ and LRP5/6 results in the disruption of the APC/Axin/GSK3 complex that is required for the targeted degradation of β-catenin.

In conclusion, we have reported a novel and a previously known sequence variant in the *WNT10B* gene in four consanguineous families affected by SHFM. This further extended the spectrum of mutations in the *WNT10B* gene that result in split hand/foot malformation. These findings can improve diagnosis and genetic counseling, and form the basis of prenatal testing.

## References

[B1] Aziz A, Khan S, Zimri FK, Muhammad N, Rashid S, Ahmad W (2014). Novel homozygous mutations in the WNT10B gene underlying
autosomal recessive split hand/foot malformation in three consanguineous
families. Gene.

[B2] Blattner A, Huber AR, Rothlisberger B (2010). Homozygous nonsense mutation in *WNT10B* and
sporadic split-hand/foot malformation (SHFM) with autosomal recessive
inheritance. Am J Med Genet A.

[B3] Boles RG, Pober BR, Gibson LH, Willis CR, McGrath J, Roberts DJ, Yang-Feng TL (1995). Deletion of chromosome 2q24-q31 causes characteristic digital
anomalies: case report and review. Am J Med Genet.

[B4] Duijf PH, Bokhoven H van, Brunner HG (2003). Pathogenesis of split-hand/split-foot
malformation. Hum Mol Genet.

[B5] Elliott AM, Evans JA (2006). Genotype-phenotype correlations in mapped split hand foot
malformation (SHFM) patients. Am J Med Genet.

[B6] Elliott AM, Evans JA (2008). The association of split hand foot malformation (SHFM) and
congenital heart defects. Birth Defects Res.

[B7] Faiyaz-Ul-Haque M, Zaidi SH, King LM, Haque S, Patel M, Ahmad M, Siddique T, Ahmad W, Tsui LC, Cohn DH (2005). Fine mapping of the X-linked split-hand/split-foot malformation
(SHFM2) locus to a 5.1-Mb region on Xq26.3 and analysis of candidate
genes. Clin Genet.

[B8] Goodman FR, Majewski F, Collins AL, Scambler PJ (2002). A 117 kb microdeletion removing HOXD9, HOXD13 and EVX2 causes
synpolydactyly. Am J Hum Genet.

[B9] Gordon MD, Nusse R (2006). Wnt signaling: Multiple pathways, multiple receptors, and
multiple transcription factors. J Biol Chem.

[B10] Gurnett CA, Dobbs MB, Nordsieck EJ, Keppel C, Goldfarb CA, Morcuende JA, Bowcock AM (2006). Evidence for an additional locus for split hand/foot malformation
in chromosome region 8q21.11-q22.3. Am J Med Genet A.

[B11] Gurrieri F, Prinos P, Tackels D, Kilpatrick MW, Allanson J, Genuardi M, Vuckov A, Nanni L, Sangiorgi E, Garofalo G (1996). A split hand-split foot (SHFM3) gene is located at
10q24-25. Am J Med Genet.

[B12] Ianakiev P, Kilpatrick MW, Toudjarska I, Basel D, Beighton P, Tsipouras P (2000). Split-hand/split-foot malformation is caused by mutations in the
p63 gene on 3q27. Am J Hum Genet.

[B13] Khan S, Basit S, Zimri FK, Ali N, Ali G, Ansar M, Ahmad W (2012). A novel homozygous missense mutation in *WNT10B*
in familial split-hand/foot malformation. Clin Genet.

[B14] Maquat LE (1996). Defects in RNA splicing and the consequence of shortened
translational reading frames. Am J Hum Genet.

[B15] Maquat LE (2005). Nonsense-mediated mRNA decay in mammals. J Cell Sci.

[B16] Matise TC, Chen F, Chen W (2007). A second-generation combined linkage physical map of the human
genome. Genome Res.

[B17] McKusick VA (1998). Mendelian inheritance in man. A catalog of human genes and
geneticdisorders.

[B18] Nunes ME, Schutt G, Kapur RP, Luthardt F, Kukolich M, Byers P, Evans JP (1995). A second autosomal split hand/split foot locus maps to chromosome
10q24-q25. Hum Mol Genet.

[B19] Ozen RS, Baysal BE, Devlin B, Farr JE, Gorry M, Ehrlich GD, Richard CW (1999). Fine mapping of the split-hand/split-foot locus (SHFM3) at 10q24:
Evidence for anticipation and segregation distortion. Am J Hum Genet.

[B20] Raas-Rothschild A, Manouvrier S, Gonzales M, Farriaux JP, Lyonnet S, Munnich A (1996). Refined mapping of a gene for split hand-split foot malformation
(SHFM3) on chromosome 10q25. J Med Genet.

[B21] Scherer SW, Poorkaj P, Allen T, Kim J, Geshuri D, Nunes M, Soder S, Stephens K, Pagon RA, Patton MA (1994). Fine mapping of the autosomal dominant split hand/split foot
locus on chromosome 7 band q21.3-q22.1. Am J Hum Genet.

[B22] Shamseldin HE, Faden MA, Alashram W, Alkuraya FS (2012). Identification of a novel DLX5 mutation in a family with
autosomal recessive split hand and foot malformation. J Med Genet.

[B23] Sobel E, Lange K (1996). Descent graphs in pedigree analysis: applications to haplotyping,
location scores, and markersharing statistics. Am J Hum Genet.

[B24] Sowinska-Seidler A, Badura-Stronka M, Latos-Bielenska A, Stronka M, Jamsheer A (2014). Heterozygous *DLX5* nonsense mutation associated
with isolated split hand/foot malformation with reduced penetrance and
variable expressivity in two unrelated families. Birth Def Res A Clin Mol Teratol.

[B25] Umair M, Ullah A, Abbas S, Ahmad F, Basit S, Ahmad W (2017). First direct evidence of involvement of a homozygous
loss-of-function variant in the EPS15L1 gene underlying
split-hand/split-foot malformation. Clin Genet.

[B26] Ugur SA, Tolun A (2008). Homozygous *WNT10B* mutation and complex
inheritance in split hand/foot malformation. Hum Mol Genet.

[B27] Ullah A, Raza SI, Ali RH, Naveed AK, Jan A, Rizvi SDA, Satti R, Ahmad W (2015). A novel deletion mutation in the *DSG4* gene
underlies autosomal recessive hypotrichosis with variable phenotype in two
unrelated consanguineous families. Clin Exp Dermatol.

[B28] Ullah A, Ullah MF, Khalid ZM, Ahmad W (2016a). A novel heterozygous frameshift mutation in *DLX5*
gene underlies isolated split hand foot malformation type 1. Pediatr Int.

[B29] Ullah A, Hammid A, Umair M, Ahmad W (2016b). A novel heterozygous intragenic sequence variant in DLX6 probably
underlies first case of autosomal dominant split-hand/foot malformation type
1. Mol Syndromol.

